# The Influence of Iron Ions on Optical Brighteners and Their Application to Cotton Fabrics

**DOI:** 10.3390/ma14174995

**Published:** 2021-09-01

**Authors:** Tihana Dekanić, Tanja Pušić, Ivo Soljačić, Branka Vojnović, Julija Volmajer Valh

**Affiliations:** 1 Department of Textile Chemistry and Ecology, Faculty of Textile Technology, University of Zagreb, HR-10000 Zagreb, Croatia; tihana.dekanic@ttf.unizg.hr (T.D.); tanja.pusic@ttf.unizg.hr (T.P.); ivo.soljacic@ttf.unizg.hr (I.S.); 2 Department of Applied Chemistry, Faculty of Textile Technology, University of Zagreb, HR-10000 Zagreb, Croatia; 3 Institute of Engineering Materials and Design, Faculty of Mechanical Engineering, University of Maribor, SI-2000 Maribor, Slovenia; julija.volmajer@um.si

**Keywords:** optical brighteners, solutions, iron ions, absorption, fluorescence, cotton fabric, whiteness degree, Ultraviolet Protection Factor (UPF)

## Abstract

The influence of iron ions at concentrations of 0.2, 0.5, and 1.0 g/L on optical brighteners of the groups stilbene and biphenyl in solution and on cotton fabric was investigated. Both groups of optical brighteners are intended for detergent formulations. The influence of iron ions was studied by absorption and fluorescence spectra in solution and by whiteness degree, identifying color differences using CIEL*a*b* coordinates and Ultraviolet Protection Factor (UPF) of cotton fabrics. The obtained results in solutions and cotton fabrics showed different behavior of optical brighteners stilbene and biphenyl in the presence of iron. Stilbene compounds with metal ions produced new species capable of absorbing in the UV-B region of the spectrum. A biphenyl compound in combination with iron had no effect on the absorption properties. Both optical brighteners were influenced by iron ions in the sense of fluorescence quenching. The influence of iron ions in single- and two-bath treatments of cotton fabrics after one cycle on whiteness degree and UPF was negligible.

## 1. Introduction

Optical brighteners (OBs) are special compounds that contain a fluorescent system instead of a chromophore and are intended for optical and UV protection applications [1]. The basic requirement for these compounds is the ability to absorb electromagnetic radiation (EMR) in the UV spectrum. It is usually associated with the molecules with planar configuration (e.g., metal complexes), conjugated double bonds, or high resonance stability, outstanding among them are aromatic compounds with an electron donor group (e.g., –NH_2_) or acidic protons (phenols and anilines) [2]. Optical brighteners absorb maximally in the near UV, from 340 to 380 nm, and emit in the visible part of the spectrum at 425 to 450 nm [3]. From a chemical point of view, OBs can be derivatives of stilbene, coumarin, benzoxazole, benzo[b]furan, etc. [4,5].

Cotton, an important biopolymer in the manufacture of woven and knitted fabrics, cannot provide adequate protection against UV radiation. UV protection of cotton textiles can be improved by fabric weaving construction parameters and finishes [6,7,8,9,10,11,12,13,14]. OBs are known finishing additives that can improve the UV protection of cotton textiles [15,16,17]. Washing such treated cotton materials with detergents without OBs leads to a loss of optical and UV protection properties. According to previous studies, UPF reaches the level of untreated fabric after 10 washing cycles [17,18]. The role of OBs in detergent formulation for washing cotton fabrics is to improve the optical and UV properties. Previous research has mainly focused on the optical properties of OBs in detergents but not on the UV properties [3,19,20,21]. OBs in detergents can provide UV protection properties as an added value of textiles in the washing process [18,22,23,24,25,26]. Stilbene and biphenyl derivatives are most commonly used as detergent OBs [3,19,20]. Such washing effects can be blocked by the presence of chlorine and various types of metal ions, depending on their concentration and composition. They can reduce the absorption and/or quench fluorescence and reduce whiteness [3,27,28,29,30]. The reason Fe ions reduce the fluorescence intensity of the OB is most likely due to impact of the Fe ion on the electronically excited molecule followed by a reverse electron-transfer reaction, which leads to the system returning to its original unexcited state. To the best of our knowledge, the effect of detergent with OBs in the presence of metal ions on UV protection properties has not been previously researched.

The importance of UV protection of cotton materials when washed with the detergents containing OBs was the motivation for the present study. There are many studies in which UV protection of cotton materials has been improved by fabric weaving construction parameters and finishes. OBs in detergents can also increase the UV protection properties as an added value of textiles in the washing process. In this study, the basis was to research the ability of the UV protection of the cotton materials when washed with the detergent containing optical brighteners (OBs) and in the presence of fluorescence quencher. For that purpose, in a first step, aqueous solutions of stilbene and biphenyl OBs were analyzed under the influence of iron ions using absorption and fluorescence spectra. The application of OBs in the presence and absence of iron ions was carried out on standard cotton fabrics by single- and two-bath treatments. The optical and protective properties of cotton fabrics before and after treatments were characterized by whiteness (W_CIE_), spectral parameters (a* and b*), and by Ultraviolet Protection Factor (UPF).

## 2. Materials and Methods

### 2.1. Materials

The chemical structures of the OBs with stilbene (S) and biphenyl structures (B) structures are shown in Table 1.

Analytical evaluation of the stilbene and biphenyl OBs in solutions with concentrations of 0.08%, 0.12%, and 0.25% was carried out. Table 2 shows the labeling of OBs with different concentrations.

The iron standard solution Fe(NO_3_)_3_ in HNO_3_ 0.5 mol/L 1000 mg/L Fe Certipur^®^ from Merck served as the source of iron ions. Appropriate aliquots were taken by diluting the stock solution in distilled water depending on the desired addition of iron ions.

Concentrations of 0.2, 0.5, and 1.0 mg/L of the iron standard solution were added to the OBs, as shown in Table 3.

The application of OBs was realized by the washing process of a cotton fabric in plain weave with the surface mass of 175.6 g/m^2^ and the density in warp/weft direction 25/25 yarns/cm. An ECE Color fastness Test Detergent 77 (5 g/L) without OBs in its composition was used [31].

### 2.2. Treatments

The treatments of cotton fabrics were carried out in a Linitest apparatus, Original Hanau as follows:

Single-bath treatment—simultaneous washing of cotton fabrics with 0.2 mg/L, 0.5 mg/L, and 1.0 mg/L iron standard solutions added to ECE reference detergent (5 g/L) with stilbene and biphenyl OBs (0.08%, 0.12%, and 0.25%) at 60 °C, bath ratio 1:20, duration 30 min.

Two-bath treatment—cotton fabrics were treated with iron ions at concentrations of 0.2, 0.5, and 1.0 mg/L (at 60 °C, bath ratio 1:20, duration for 30 min). Samples were rinsed with water and washed with ECE reference detergent containing stilbene and biphenyl OBs (0.08%, 0.12%, and 0.25%) at 60 °C, bath ratio 1:20, for 30 min.

Treated cotton samples specially marked with asterisks, * for single-bath treatment and ** for two-bath treatment, were dried under ambient conditions and evaluated objectively by methods.

### 2.3. Methods

UV/Vis spectrophotometer Lambda 20, Perkin Elmer, was used under the following measurement conditions: range from 200 to 500 nm, aperture size 2 nm, and reading-off range 1 nm for absorption spectra of solutions. Fluorimeter F-7000, Hitachi (Tokyo, Japan), was used to measure fluorescence intensity in the wavelength range of 300 to 600 nm, at excitation wavelength of 250 nm, and wavelength scan speed of 240 nm/min.

The spectral characteristics of cotton fabrics before and after treatments were measured using a Spectraflash SF 600+ CT, Datacolor, remission spectrophotometer in the measurement range of 360 to 700 nm, with an aperture of 20 mm, standard illumination D65, and reported as the mean of three individual measurements. The W_CIE_ was calculated automatically, according to EN ISO 105-J02 [32]. Transmittance was measured with a UV/V is spectrophotometer Varian-Cary 50/Solascreen, using a software package to calculate specific *UPF* values using the in vitro method according to AS/NZS 4399:1996 [33,34]. *UPF* is an expression indicating the degree of protection against UV radiation and is calculated automatically from the transmittance *T*(*λ*), according to the following equation:

(1) $$UPF=\frac{\sum_{\lambda =290}^{400}E(\lambda )\cdot \varepsilon (\lambda )\cdot \Delta \lambda }{\sum_{\lambda =290}^{400}E(\lambda )\cdot T(\lambda )\cdot \varepsilon (\lambda )\cdot \Delta \lambda }$$

where:

*E*(*λ*)—relative erythemal spectral effectiveness (W m^−2^ nm^−1^);

*ε*(*λ*)—solar spectral irradiance;

Δ*λ*—measured wavelength interval (nm);

*T*(*λ*)—average spectral transmittance of the specimen (%).

## 3. Results and Discussion

**Figure 1:**
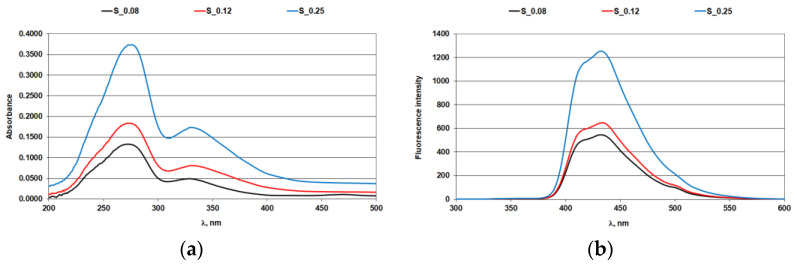
Stilbene optical brightener: (**a**) absorption spectra; (**b**) fluorescence spectra.

**Figure 2:**
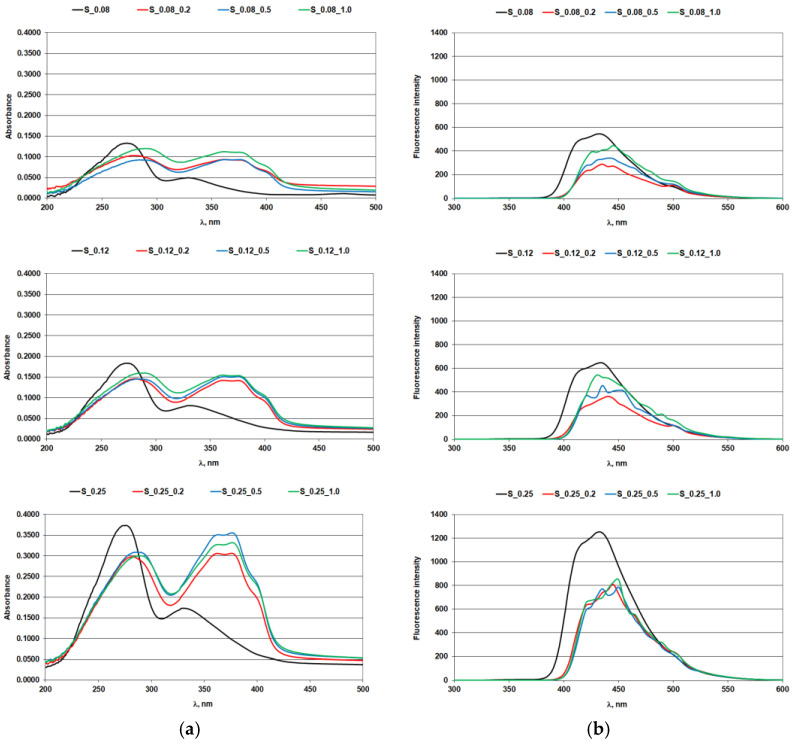
Stilbene optical brightener with iron ions: (**a**) absorption spectra; (**b**) fluorescence spectra.

**Figure 3:**
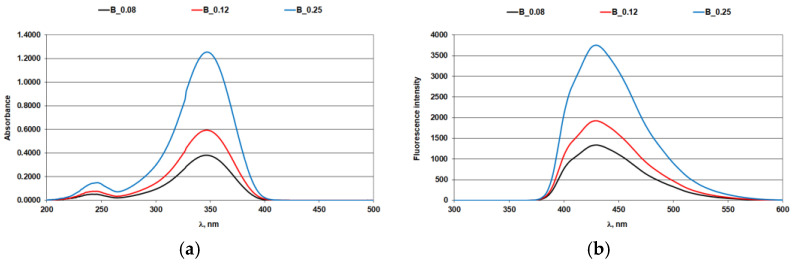
Biphenyl optical brightener: (**a**) absorption spectra; (**b**) fluorescence spectra.

**Figure 4:**
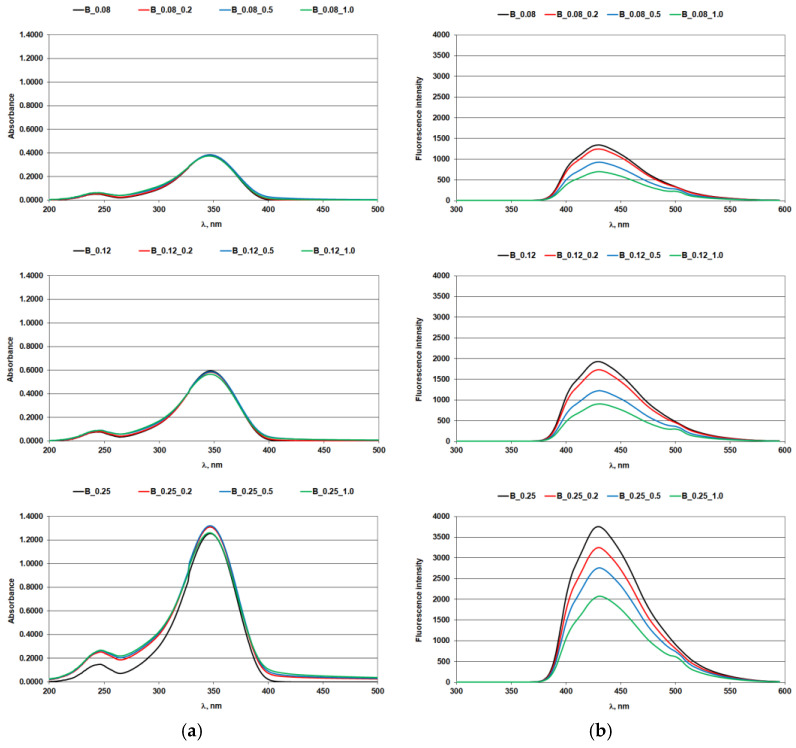
Biphenyl optical brightener with iron ions: (**a**) absorption spectra; (**b**) fluorescence spectra.

The effect of iron ions on the optical brighteners in solution was assessed using absorption and fluorescence spectra (Figure 1, Figure 2, Figure 3 and Figure 4; Table 4 and Table 5).

**Figure 5:**
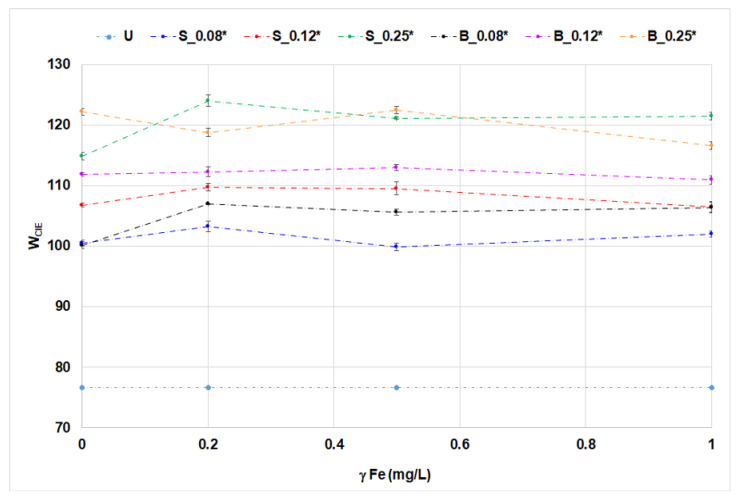
Whiteness degree (W_CIE_) of cotton samples treated in single-bath treatment.

**Figure 6:**
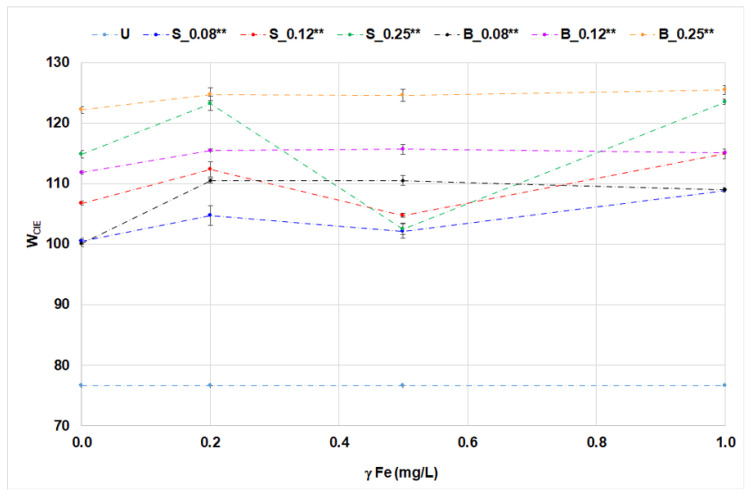
Whiteness degree (W_CIE_) of cotton samples treated in two-bath treatment.

**Figure 7:**
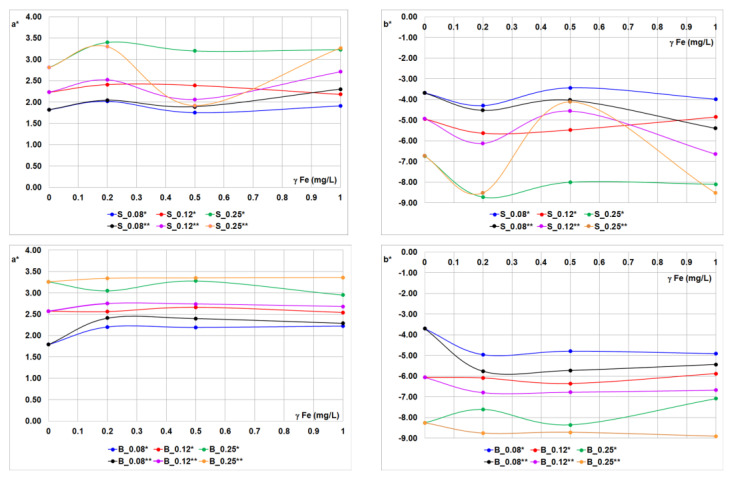
Spectral coordinates, a* and b*, of cotton fabrics treated in a single-bath and two-bath method.

**Figure 8:**
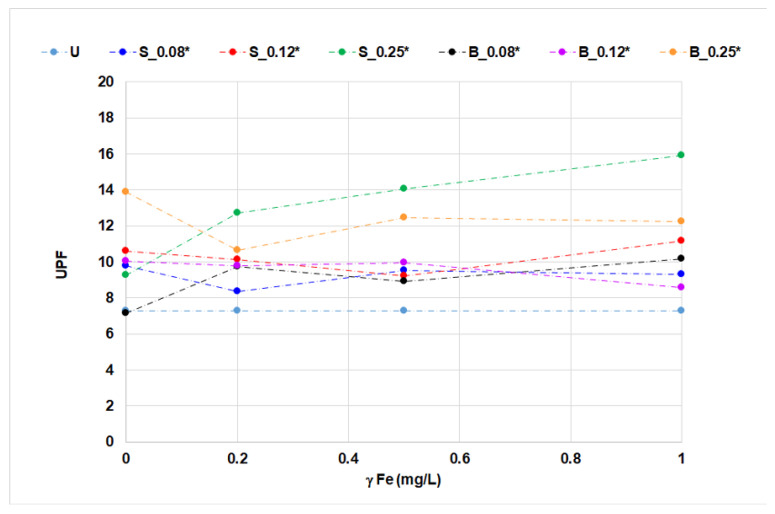
UPF of cotton fabrics treated in single-bath treatment.

**Figure 9:**
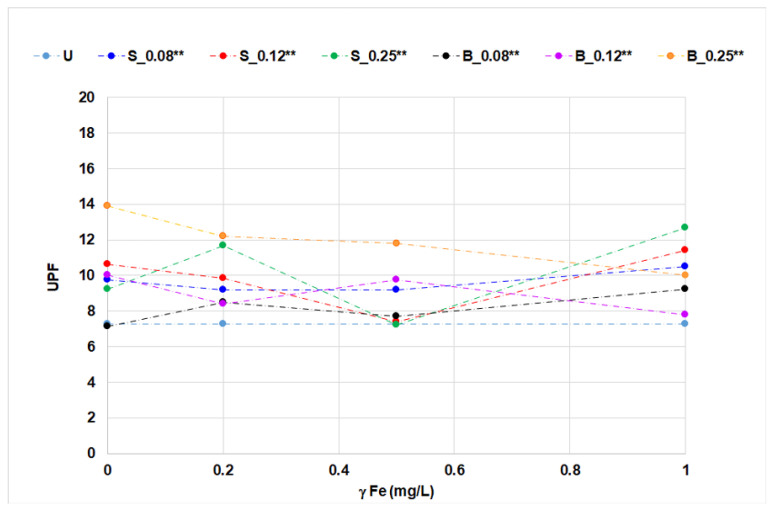
UPF of cotton fabrics treated in two-bath treatments.

The application of OBs with iron ions on cotton fabrics was analyzed by whiteness degree, CIEL*a*b* coordinates, and UPF (Figure 5, Figure 6, Figure 7, Figure 8 and Figure 9, Table 6).

### 3.1. Absorption and Fluorescence Curves of Optical Brighteners in a Solution

In Figure 1a,b, the absorbance and fluorescence spectra of stilbene OB are plotted for three different concentration ranges. The results obtained showed that increased concentration of optical brightener resulted in higher absorption of UV radiation (S-0.08 < S-0.12 < S-0.25), Figure 1a. The stilbene optical brightener exhibits two absorption peaks, one in the UV-A (at wavelength 330 nm) region and one in the UV-C (at wavelength 273 nm) region. The fluorescence maximum of stilbene OB is at 430 nm (Figure 1b).

Figure 2 shows the comparison of the absorption and fluorescence spectra of the optical brighteners used on stilbenes as a function of the concentration of iron ions. The absorption spectra of stilbene OB at three concentrations after addition of ferrous ions show a bathochromic and hypochromic shift. There was a bathochromic shift of the absorption maximum (Figure 2a; Table 4) to higher wavelengths, i.e., to the UV-A (375 nm) and UV-B (286 nm) regions, which is particularly evident at higher concentrations of optical brighteners. The highest hypochromic shift was registered at the concentration of iron ions 0.5 mg/L by the absorption maximum (Figure 2a).

The iron ions and the optical brightener stilbene (Figure 2a; Table 4) generated a different species compared to the parent compound (at about 360 nm) that absorbs in the UV-A region, just like a OB. These likely new complexes open up a platform for original investigative questions. 

In spite of the fact that the mechanism of fluorescence quenching (or reduction of fluorescence intensity) has not been investigated in this paper, it can be seen from the shape of the fluorescence curve that quenching occurred most probably due to the formation of a complex between iron ions and OB. This newly formed complex had less pronounced fluorescent properties, which is manifested in a decrease in fluorescence intensity (Figure 2b). This effect was more pronounced with lower concentrations of OB, which were still sufficient to form a complex with iron presented in the solution. On the other hand, the shape of the fluorescence curve in Figure 2b shows that the lowest concentrations of iron (0.2 mg/L) with a lower concentration of OB (0.12%) caused the largest decrease in fluorescence intensity, and the shape of the curve is identical to the curve of OB but with lower fluorescence intensity. It is possible that further increasing the concentration of iron, precisely because of its excess, complexes with a different ratio of Fe:OB were formed, which can be noticed from the slightly changed shape of the curve. Definitely, in further research, it is necessary to determine the mechanism of this reaction in order to confirm these effects. Stilbene OBs contain a double bond in their molecular structure, which can exist in cis and trans isomer. Exposure to sunlight can result in a decrease or loss of their fluorescence due to cis/trans isomerization [35], with the trans isomer fluorescing strongly while the cis isomer has no fluorescence. The absorption peaks of the cis isomer are much stronger. Their fluorescence emission peaks are in the range of 400–650 nm [36].

Since UV-C rays of the sunlight spectrum do not reach the Earth’s surface [32,33,34,35], only UV-A and UV-B components of the sunlight spectrum are important for protective phenomena. The absorption and fluorescence spectra of biphenyl OB for three different concentration ranges are shown in Figure 3. The optical brightener biphenyl exhibits two absorption peaks, one in the UV-A region (at a wavelength of 347 nm) and one in the UV-C region (at a wavelength of 246 nm), Figure 3a. The peak of the fluorescence maximum of biphenyl OB is at 430 nm (Figure 3b).

Iron ions at the concentrations used did not affect the absorption characteristics of biphenyl OB in the UV-A region of the spectrum, confirming its stability in this region, (Figure 4a and Table 5). Despite the absorption stability of biphenyl OB being comparable to that of stilbene OB, the iron ion caused a quench in the fluorescence of both derivatives, Figure 2b and Figure 4b.

### 3.2. Whiteness Degree and Spectral a* and *b* Coordinates of Cotton Fabric

In the treatment of cotton fabrics, the influence of iron ions on optical effects and UV protection effects was additionally investigated. The values of whiteness degree for cotton fabrics treated with single-bath treatment can be seen in Figure 5 and Figure 6.

Whiteness degree increased with an increasing concentration of OBs, as expected. The influence of iron ions on the whiteness of cotton fabrics depends on OB derivative and treatments. Their influence in solutions of OBs is observed by fluorescence quenching (Figure 2b and Figure 4b). This phenomenon can be explained by the fact that iron ions cause fluorescence quenching, which is more pronounced in solutions than on a textile fabric. When treated with one bath, the differences in whiteness were less pronounced than when treated with two-bath treatment. Obviously, metal ions in a single-bath procedure created complexes with the molecules of stilbene optical brightener and reduced its fluorescence potential. A dramatic decrease of the whiteness of S_0.25 at 0.5 mg/L Fe and then increase at 1 mg/L Fe is specific to the two-bath treatment. The reason for the decrease is a specific and stable interaction of 0.5 mg/L Fe with cotton fabric. The concentration of 1 mg/L Fe caused less effect because it was adsorbed and desorbed through movement in a Linitest apparatus. Biphenyl OB is more stable according to the proposed evaluation criteria.

The effect of iron ions added to the fluorescent compounds on washed cotton fabrics was monitored using the CIEL*a*b* color space lightness in Table 6 and a*b* coordinates, Figure 7.

The addition of iron ions to OBs in single- and two-bath treatments did not affect the lightness of cotton fabrics (Table 6). Optical brighteners applied at higher concentrations, in interacting with iron ions, altered the a* and b* values. The changes were more pronounced in two-bath treatment. The trend was more evident with stilbene OB at the concentration of 0.25% *w*/*w* and 0.5 mg/L iron ions (Figure 7).

Iron ions influenced the negative value of b* coordinate of treated cotton fabric. This phenomenon can be explained by the formation of complex between iron ions and OB stilbene type, since the newly formed complex had less pronounced fluorescent properties, visible as a decrease in fluorescence intensity (Figure 2b). The effect was more pronounced with lower concentrations of OB. Furthermore, the iron ion solution was slightly pale-yellow-colored, which is obviously sufficient to alter the fabric hue. Due to the present yellowish hue of the iron solution, it strongly affected the -b* coordinate at lower OBs concentrations and in two-bath treatment. Namely, the a two-bath treatment, the cotton fabric was treated with iron ions first, followed by washing in a detergent solution containing OB. In this case, the iron ions were bound on cotton fabric, so the washing process with a low amount of OBs was not sufficient to neutralize the fabric chromacity. It was a somewhat different situation in the case of the single-bath treatment. The impact was less pronounced in a single bath due to the formation of a complex with a different ratio of Fe:OB, and quenching occurred.

### 3.3. UPF

The equation for calculating the UPF clearly shows that the UV-C part of the spectrum (radiation of the wavelengths below 280 nm) is not included, since this radiation does not reach the earth’s surface. The absorption results (Table 4 and Table 5) for the OBs solutions show that biphenyl and stilbene derivative absorb in the UV-A and UV-C. The addition of iron ions changes the absorption spectra of stilbene OB. The absorption maximum of UV-C was shifted to higher wavelengths, to UV-B, Table 4.

Untreated cotton fabrics exhibited a negligible degree of protection (UPF 7.276), mainly due to their structural properties and prebleaching processing level [7]. According to the influence of iron ions on stilbene OB in solution, expressed by the complex form, it was expected that the UPF of treated cotton fabrics would be increased. The UPF results of cotton fabric after single- and two-bath treatments were only slightly increased despite differences in absorption and fluorescence. The highest UV protection (UPF 15.923) was observed on cotton fabric after single-bath treatment with stilbene OB at 0.25% concentration with the highest concentration of iron ions (1.0 mg/L). Two-bath treatment, especially at 0.25% *w*/*w*, reduced the UPF, which may be attributed to the ion-exchange properties of cotton cellulose and its reaction with iron ions (Figure 8 and Figure 9).

The impact of iron ions on UPF at single- or two-bath treatments is not easy to analyze unambiguously. The results presented for the change of shade in cotton fabrics treated with fluorescent compounds with the presence of metal ions can also have an impact on the UV protection properties, as fabric chromacity (presence of a color, Figure 7) results in higher UPF values. In contrast to stilbene OB, two-bath treatment of cotton fabric with biphenyl OB at higher concentrations of iron ions slightly reduced the UPF value. The equation used to calculate UPF clearly shows that the UV-C part of the spectrum (radiation of the wavelengths below 280 nm) is not included, as this radiation does not reach the Earth surface. Absorption results (Table 5) for the florescent compound solutions show that biphenyl OB absorbs in this part of the spectrum as well. This fact is directly reflected in the lower UPF values of the fabric treated with a biphenyl derivative.

## 4. Conclusions

The influence of iron ions on optical brighteners in a solution and on treated cotton fabric was studied by analyzing of optical and protective phenomena. Two parameters, absorption and fluorescence spectra of optical brighteners in a solution, were compared with three parameters on cotton fabric: whiteness degree, hue changes, and UPF.

The results obtained showed that iron ions with stilbene as optical brightener produce a different species that absorbs in the UV-B region compared to the parent compound. It can be assumed that it is a new complex form between the stilbene optical brighteners and the iron ions.

In contrast, in the case of the biphenyl optical brightener, the iron ions had no effect on the absorption characteristics in the UV-A part of the spectrum, confirming its stability in this region. However, the influence of iron ions is noticeable by an increased absorption of the biphenyl optical brightener in the UV-C region.

The addition of iron ions in solution reduced the fluorescence intensity of the optical brighteners stilbene and biphenyl at all applied concentrations. The effect of iron ions on the absorption spectra of stilbene optical brightener in the form of bathochromic shift to the UV-C region, and on the fluorescence spectra of stilbene and biphenyl derivatives in the forming of fluorescence, quenching was observed.

The values of whiteness degree for cotton fabrics treated with a fluorescent compound with the addition of iron ions are generally lower in the case of the single-bath treatment. It has been proved that the metal ions form complex with the molecules of the optical brightener stilbene during one bath treatment, thereby reducing its potential.

The obtained results proved that the absorption and fluorescence as optical behavior in the solutions cannot be fully transferred to the optical and protective properties of the cotton fabrics obtained in single- and two-bath treatments with the detergent containing optical brighteners. We suggest that the contribution of the detergent partly caused a dispersive composition in which the influence of iron ions was suppressed.

## Figures and Tables

**Table 1:** Chemical structure of the fluorescent compounds.

Label	Name	Structural Formula	Formula	Molecular Weight (g/mol)
S	disodium 4.4′-bis[(4-anylino-6-morpholino-1.3.5-triazine-2-yl)amino]-stilbene-2.2′-disulphonate	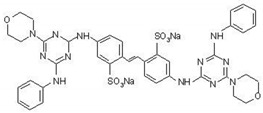	C_40_H_38_N_12_O_8_S_2_Na_2_	924.93
B	4.4′-bis(2-sulfostyryl)biphenyl	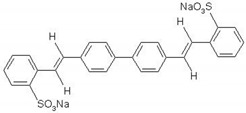	C_28_H_22_O_6_S_2_Na_2_	518.60

**Table 2:** Labeling of the optical brighteners solutions.

OBs Derivative	w_OB_ (%)	Label
Stilbene	0.08	S-0.08
	0.12	S-0.12
	0.25	S-0.25
	0.08	B-0.08
Biphenyl	0.12	B-0.12
	0.25	B-0.25

**Table 3:** Labelling of OBs with iron ions.

	Label			
γ _Fe_ (mg/L)	0	0.2	0.5	1.0
w_S_ (%)	S-0.08	S-0.08_0.2	S-0.08_0.5	S-0.08_1.0
	S-0.12	S-0.12_0.2	S-0.12_0.5	S-0.12_1.0
	S-0.25	S-0.25_0.2	S-0.25_0.5	S-0.25_1.0
w_B_ (%)	B-0.08	B-0.08_0.2	B-0.08_0.5	B-0.08_1.0
	B-0.12	B-0.12_0.2	B-0.12_0.5	B-0.12_1.0
	B-0.25	B-0.25_0.2	B-0.25_0.5	B-0.25_1.0

**Table 4:** Wavelength values at peak absorption of stilbene optical brightener solution with iron ion, as dependent upon the UV region of absorption.

Label	Spectrum		
	UV-A (315–400)	UV-B (280–315)	UV-C (100–280)
		λ_max_	
S-0.08	330	-	273
S-0.08_0.2	375	286	-
S-0.08_0.5	375	286	-
S-0.08_1.0	375	286	-
S-0.12	330	-	273
S-0.12_0.2	375	286	-
S-0.12_0.5	375	286	-
S-0.12_1.0	375	286	-
S-0.25	330	-	273
S-0.25_0.2	375	286	-
S-0.25_0.5	375	286	-
S-0.25_1.0	375	286	-

**Table 5:** Wavelength values at peak absorption of biphenyl optical brightener solution with iron ion, as dependent upon the UV region of absorption.

Label	Spectrum		
	UV-A (315–400)	UV-B (280–315)	UV-C (100–280 nm)
λ_max_			
B-0.08	347	-	246
B-0.08_0.2	347	-	246
B-0.08_0.5	347	-	246
B-0.08_1.0	347	-	246
B-0.12	347	-	246
B-0.12_0.2	347	-	246
B-0.12_0.5	347	-	246
B-0.12_1.0	347	-	246
B-0.25	347	-	246
B-0.25_0.2	347	-	246
B-0.25_0.5	347	-	246
B-0.25_1.0	347	-	246

**Table 6:** Lightness of cotton fabric samples treated with iron ions.

Cotton Fabrics Treatment	γ_Fe_ (mg/L)			
Single-bath	0	0.2	0.5	1.0
S_0.08*	93.23	93.23	93.49	93.31
S_0.12*	93.52	93.42	93.68	93.61
S_0.25*	93.47	93.57	93.75	93.72
B_0.08*	93.06	93.56	93.31	93.41
B_0.12*	93.51	93.66	93.39	93.46
B_0.25*	93.70	93.45	93.64	93.54
**Cotton Fabrics Treatment**	**γ_Fe_ (mg/L)**			
Two-bath	0	0.2	0.5	1.0
S_0.08**	93.23	93.42	93.28	93.53
S_0.12**	93.52	93.61	93.35	93.70
S_0.25**	93.47	93.67	93.27	93.77
B_0.08**	93.06	93.51	93.59	93.51
B_0.12**	93.51	93.67	93.80	93.70
B_0.25**	93.70	93.78	93.92	93.91
Untreated	93.60	93.60	93.60	93.60

## Data Availability

Data available in a publicly accessible repository.

## References

[1] Mustalish R.A. (2000). Optical brighteners: History and technology. Stud. Conserv..

[2] Skoog D.A., Holler F.J., Crouch S.R., Skoog D.A., Douglas A., Skoog F., James H., Stanley R.C. (2007). Principles of Instrumental Analysis.

[3] Shore J., Shore J. (2002). Fluorescent brightening agent. Colorants and Auxiliaries: Organic Chemistry and Application Properties.

[4] Tiki A., Amin A., Kanwal A. (2010). Chemistry of optical brighteners and uses in textile industries. Pak. Text. J..

[5] Bayly A.E., Smith D.J., Roberts N.S., York D.W., Capeci S., Zoller U., Sosis P. (2009). Detergent Processing. Handbook of Detergents, Part F: Production.

[6] Kostajnšek K., Dimitrovski K. (2021). Use of Extended Cover Factor Theory in UV Protection of Woven Fabric. Polymers.

[7] Zimniewska M., Batog J., Kozlowski R.M. (2012). Ultraviolet-blocking properties of natural fibres. Handbook of Natural Fibres.

[8] Hoffmann K., Kaspar K., Gambichler T., Altmeyer P. (2000). In vitro and in vivo determination of the UV protection factor for lightweight cotton and viscose summer fabrics: A preliminary study. J. Am. Acad. Dermatol..

[9] Zhu J., Li H., Wang Y., Wang Y., Yan J. (2021). Preparation of Ag NPs and Its Multifunctional Finishing for Cotton Fabric. Polymers.

[10] Urbas R., Kostajnšek K., Dimitrovski K. (2011). Impact of structure and yarn colour on UV properties and air permeability of multilayer cotton woven fabrics. Text. Res. J..

[11] Dobnik Dubrovski P., Golob D. (2009). Effects of Woven Fabric Construction and Color on Ultraviolet Protection. Text. Res. J..

[12] Stanković S.B., Popović D., Poparić G.B., Bizjak M. (2009). Ultraviolet Protection Factor of Gray-state Plain Cotton Knitted Fabrics. Text. Res. J..

[13] Achwal W.B. (2000). UV Protection by Textiles. Colourage.

[14] Pezelj E., Tomljenović A., Čunko R. (2004). Textiles for the Protection against Sun Radiation. Tekstil.

[15] Cox Crews P., Katchman S., Beyer A. (1999). Influences on UVR Transmission of Undyed Woven Fabrics. Text. Chem. Colorists.

[16] Kim J., Stone J., Crews P., Shelley M., Hatch K.L. (2004). Improving Knit Fabric UPF Using Consumer Laundry Products: A Comparison of Results Using Two Instruments. Fam. Consum. Sci. Res. J..

[17] Bajaj P., Kothari V.K., Ghosh S.B. (2000). Some innovations in UV protective clothing. Indian J. Fibres Text. Res..

[18] Eckhardt C., Rohwer H. (2000). UV protector for cotton fabrics. Text. Chem. Colorist Am. Dyest. Report..

[19] Heinemann G.W., Merkle G., Waldhoff H., Spilker R. (2005). Determination of Optical Brighteners in Laundry Detergents. Handbook of Detergents, Part C, Analysis.

[20] Zappone M., Kaziska A., Bogush G., Zoller U. (2009). Applications of detergent in laundering. Handbook of Detergents, Part E: Applications.

[21] Salas H., Gutiérrez-Bouzán C., López-Grimau V., Vilaseca M. (2019). Respirometric Study of Optical Brighteners in Textile Wastewater. Materials.

[22] Saravanan D. (2007). UV protection textile materials. AUTEX Res. J..

[23] Stanford D.G., Georgouras K.E., Pailthorpe M.T. (1995). Sun protection afforded by a summer weight garment: Effect of wash and wear. Med. J. Aust..

[24] Stanford D.G., Georgouras K.E., Pailthorpe M.T. (1995). The effect of laundering on the sun protection afforded by a summer weight garment. J. Eur. Acad. Dermatol. Venereol..

[25] Gies P. (2007). Photoprotection by clothing, Photodermatology. Photoimmunol. Photomed..

[26] Sarkar A.K. (2007). On the relationship between fabric processing and ultraviolet radiation transmission. Photodermatol. Photoimmunol. Photomed..

[27] Soljačić I., Čunko R. (1979). Wirkung von Kupfer-und Eisensalzen auf die Weißeffekte optisch aufgehellter Baumwolle. Melliand Text..

[28] Milligan B., Holt L. (1974). Fluorescent whitening agents I Bis-4,4’-(4’’-methoxy-6’’-phenoxy-s-triazin-2’’-ylamino)stilbene-2,2’-disulphonic acid: Its photodecomposition in solution and on wool. Aust. J. Chem..

[29] Blanco M., Jiménez L., Valverde I. (2001). Stability of a Stilbene-Type Fluorescent Whitening Agent against Hypochlorite. Text. Res. J..

[30] Mainali B., Pham T.T., Ngo H.H., Guo W. (2013). Maximum allowable values of the heavy metals in recycled water for household laundry. Sci. Total Environ..

[31] https://www.farbechtheit.info/pdfs/sicherheitsdatenblaetter/DEK_ECE77_E.PDF.

[32] (2000). EN ISO 105-J02:2000: Textiles—Tests for Colour Fastness—Part J02: Instrumental Assessment of Relative Whiteness.

[33] (1996). AS/NZS 4399:1966 Standard Test Method for Sun Protective Clothing Evaluation and Classification. Standards Australian: Homebush, Australia.

[34] Chitichotpanya P., Chitichotpanya C. (2017). In Vitro Assessment of Sericin-Silver Functionalized Silk Fabrics for Enhanced UV Protection and Antibacterial Properties Using Experimental Design. Coatings.

[35] Neiditch O.W. (1981). Minor additives in heavy-duty laundry detergents. J. Am. Oil Chem. Soc. Vol..

[36] Grabchev I. (2000). Photochemistry of some polymerizable fluorescent brighteners. J. Photochem. Photobiol. A Chem..

